# Erastin, a ferroptosis-inducing agent, sensitized cancer cells to X-ray irradiation via glutathione starvation *in vitro* and *in vivo*

**DOI:** 10.1371/journal.pone.0225931

**Published:** 2019-12-04

**Authors:** Yuki Shibata, Hironobu Yasui, Kei Higashikawa, Naoki Miyamoto, Yuji Kuge

**Affiliations:** 1 Department of Biomedical Imaging, Graduate School of Biomedical Science and Engineering, Hokkaido University, Sapporo, Hokkaido, Japan; 2 Central Institute of Isotope Science, Hokkaido University, Sapporo, Hokkaido, Japan; 3 Laboratory of Radiation Biology, Department of Applied Veterinary Sciences, Graduate School of Veterinary Medicine, Hokkaido University, Sapporo, Hokkaido, Japan; 4 Faculty of Engineering, Hokkaido University, Sapporo, Hokkaido, Japan; Central Research Institute of Electric Power Industry (CRIEPI), JAPAN

## Abstract

High concentrations of antioxidants in cancer cells are huge obstacle in cancer radiotherapy. Erastin was first discovered as an inducer of iron-dependent cell death called ferroptosis accompanied by antioxidant depletion caused by cystine glutamate antiporter inhibition. Therefore, treatment with erastin is expected to potentially enhance cellular radiosensitivity. In this study, we investigated the influence of treatment with erastin on the radiation efficiency against cancers. The clonogenic ability, glutathione peroxidase 4 (GPX4) expression, and glutathione concentration were evaluated using HeLa and NCI-H1975 adenocarcinoma cell lines treated with erastin and/or X-ray irradiation. For in vivo studies, NCI-H1975 cells were transplanted in the left shoulder of nude mice, and then radiosensitizing effect of erastin and glutathione concentration in the cancer were evaluated. Treatment with erastin induced ferroptosis and decreased the concentration of glutathione and GPX4 protein expression levels in the two tumor cell lines. Moreover, erastin enhanced X-ray irradiation-induced cell death in both human tumor cell lines. Furthermore, erastin treatment of a tumor-transplanted mouse model similarly demonstrated the radiosensitizing effect and decrease in intratumoral glutathione concentration in the *in vitro* study. In conclusion, our study demonstrated the radiosensitizing effect of erastin on two adenocarcinoma cell lines and the tumor xenograft model accompanied by glutathione depletion, indicating that ferroptosis inducers that reduce glutathione concentration could be applied as a novel cancer therapy in combination with radiotherapy.

## Introduction

Iron homeostasis in cancer cells, which has been widely studied, indicates the importance of iron in tumorigenesis and tumor development [1–3]. Ferrous iron has cellular toxicity, which is expressed with the production of reactive oxygen species (ROS) through Fenton reactions. Therefore, cellular iron homeostasis is strictly regulated by iron-dependent proteins [4–6]. However, iron homeostasis is often disrupted in cancer cells, which leads to excessive iron accumulation [7], partially because that iron is essential for maintaining the aberrantly high growth rate of cancer cells by supplying the iron-dependent enzyme ribonucleotide reductase [8]. Iron transport is mainly mediated by the transferrin–transferrin receptor (TfR) complex in most cells. Several cancer cell lines express higher levels of the TfR1 protein compared to the normal cells, and the TfR1 expression level is correlated with the malignancy [9–11]. Hence, intracellular iron and TfR1 have been considered as the targets of cancer therapies [12].

As mentioned above, cancer cells have abundant amount of iron and are therefore often exposed to excessive oxidative stress. However, cancer cells produce sufficient amounts of antioxidants, such as glutathione, to protect themselves from oxidative stress [13]. Therefore, high concentrations of glutathione are a major obstacle to cancer chemotherapy and radiotherapy [14]. To overcome this therapy resistance, strategies targeting glutathione depletion have been widely investigated. For example, buthionine sulfoximine (BSO), a well known synthetic glutathione inhibitor, was reported to show a chemosensitizing effect in myeloma and neck cancers [15]. Moreover, a combination of BSO and melphalan, a nitrogen mustard alkylating agent, is used on neuroblastoma patients in clinical trials [16].

In 2012, a novel programmed cell death triggered by iron-dependent accumulation of lipid ROS, called as ferroptosis, was identified [17]. Ferroptosis is distinct from other well-known forms of cell death, such as apoptosis, necrosis, and autophagy, owing to its iron dependence. The serum iron transporter transferrin is necessary for inducing ferroptosis and the levels of TfR1 expression correlate with ferroptosis sensitivity [18, 19]. As cell death is strictly regulated by iron accumulation and antioxidant production capability of cancer cells, which are abundant in iron, ferroptosis is a useful approach to cancer therapy. Erastin, an inducer of ferroptosis, is identified as an inhibitor of cystine/glutamate antiporter (xCT) and glutathione synthesis [20]. In addition, sulfasalazine, a clinical drug for inflammatory bowel disease, is an xCT inhibitor that induces ferroptosis [17]. These drugs have an antitumor effect by ferroptosis induction [21–23]. In addition, these ferroptosis inducers can enhance the effect of chemotherapeutic agents such as cisplatin and temozolomide [24–26]. However, there are only a few studies on the efficacy of the treatment with a combination of these ferroptosis inducers and X-ray irradiation. In this study, we hypothesized that erastin modulates a ferroptosis-related pathway and affects the sensitivity of cancer cells to X-ray irradiation-induced cell death using two human cancer cell lines and tested this hypothesis *in vitro* and *in vivo*.

## Materials and methods

### Reagents

Erastin was purchased from AdooQ Bioscience (Irvine, CA) and ferrostatin-1 from Sigma-Aldrich (St. Louis, MO). The following antibodies were used for western blotting: anti-glutathione peroxidase 4 (Cat. No. ab125066, Abcam, Cambridge, UK), anti-βactin (Sigma-Aldrich), and horseradish peroxidase-conjugated secondary antibodies (Promega, Madison, WI).

### Cell culture

Human cervical adenocarcinoma cells (HeLa) and lung adenocarcinoma cells (NCI-H1975) were purchased from the RIKEN Cell Bank (Tsukuba, Japan) and American Type Culture Collection (Manassas, VA), respectively. These cells were grown in the RPMI-1640 medium (Sigma-Aldrich) supplemented with 10% fetal bovine serum (CELLect®, MP Biomedicals, Santa Ana, CA) and 100 units/mL of penicillin-streptomycin (MP Biomedicals). The cells were maintained at 37°C in 5% CO_2_.

### X-ray irradiation and drug treatment

Cells were irradiated with X-rays using a linear accelerator (CLINAC 6EX, Varian Medical Systems, Palo Alto, CA) at doses of 2.5, 5.0, 7.5, and 10 Gy (dose rate, 2.19 Gy/min). The prescribed dose was defined to be at the isocenter. HeLa and NCI-H1975 cells in 60 or 90 mm plastic dishes were allowed to adhere to the dishes at 37°C in 5% CO_2_ for 6 h. Subsequently, they were treated with erastin alone (0.1, 1, 5, 10, 20, or 50 μM) or with erastin and ferrostatin-1 (1 μM) and incubated for 24 h.

### Clonogenic survival assay

An appropriate number of tumor cells attached to 60 mm dishes were treated with the compounds and/or X-ray irradiation. After the treatment, the compounds were removed by replacing the medium with a fresh one and the cells were incubated in a humidified 5% CO_2_ atmosphere at 37°C for nine days. The cell colonies were fixed with methanol, stained with the Giemsa solution, and counted under a microscope (CKX41, Olympus, Tokyo, Japan). Only the colonies containing more than 50 cells were counted as surviving colonies. The survival curves were fit to a linear-quadratic (LQ) model using the data analysis software GraphPad Prism 7 (GraphPad Software Inc., San Diego, CA).

### Sodium dodecyl sulfate polyacryl amide gel electrophoresis (SDS-PAGE) and western blotting

The HeLa and NCI-H1975 cells were collected and lysed in a RIPA buffer (Thermo Fisher Scientific, Carlsbad, CA) with a protease inhibitor cocktail (Roche Diagnostics, Basel, Switzerland) and subjected to two freeze–thaw cycles. The lysed cells were centrifuged at 15,000×*g* for 20 min at 4°C, and the supernatants were collected as protein samples. Laemmli’s sample buffer (Bio-Rad, Hercules, CA) was added to the supernatants, and the mixture was boiled for 5 min. Proteins were separated by SDS-PAGE and transferred onto a PVDF membrane (Bio-Rad) at 60 V in a transfer buffer (25 mM Tris, 192 mM glycine, and 20% methanol) for 60 min at 4°C. The membrane was probed overnight with specific antibodies diluted with TBST (10 mM Tris-HCl [pH 7.4], 0.1 M NaCl and 0.1% Tween-20) containing 5% skim milk (Wako Pure Chemical Industries, Osaka, Japan) at 4°C. After probing with HRP-conjugated secondary antibodies, the bound antibodies were detected with an Immobilon^®^ western HRP substrate (MilliporeSigma, Burlington, MA). Densitometry was performed using Multi Gauge V3.0 software (Fujifilm, Tokyo, Japan).

### Measurement of glutathione concentration

Concentrations of the reduced (GSH) and oxidized (GSSG) forms of glutathione were determined with a GSSG/GSH quantification kit (Dojindo Laboratories, Kumamoto, Japan). The harvested cells were lysed in 10 mM HCl and 1% 5-sulfosalicylic acid dihydrate (Wako Pure Chemical Industries). The lysate was centrifuged (8,000×*g*) and the supernatant was collected. An equal volume of H_2_O was added to the supernatant and the mixture was incubated with coloring agents. The tumor tissues were lysed in 5% 5-sulfosalicylic acid dihydrate and homogenized with a bead cell disrupter MS-100R (Tomy Seiko Co., Ltd., Tokyo, Japan). After the lysate was centrifuged, the supernatant was collected and added H_2_O up to a final concentration of 0.5% 5-sulfosalicylic acid. The supernatant with 0.5% 5-sulfosalicylic acid was incubated with coloring agents as described above. The absorption of DTNB (λ_max_ = 412 nm) was measured with a multi mode plate reader PowerScan HT (DS Pharma Biomedical Co., Ltd., Osaka, Japan), and concentrations of GSH and GSSG were estimated in accordance with the manufacturer’s protocol.

### Tumor transplantation

All animal experiments were performed in accordance with the Guideline for Animal Experiments of the Graduate School of Medicine, Hokkaido University, and approved by the Laboratory Animal Care and Use Committee of Hokkaido University (Approval number 16–0102, 18–0111). The mice were housed in a 12-h light/12-h dark cycle with food and water supplied ad libitum. Female BALB/c Slc-nu/nu mice aged 8–10 weeks were purchased from Japan SLC (Hamamatsu, Japan). The NCI-H1975 cells (5 × 10^6^ cells/100 μL of Phosphate-Buffered Saline [PBS]) were inoculated subcutaneously into the left forelimbs of the mice under anesthesia induced with 2% isoflurane. The tumor size was measured using a caliper every other day from 6 days after cell inoculation and calculated as V (mm^3^) = (L × W^2^)/2, where L and W are the tumor length and width, respectively. Tumor-transplanted mice were ethically sacrificed when the tumor volume reached at 2,000 mm^3^ or a tumor burden greater than 10% of the body weight. The mice were sacrificed by cervical dislocation under 2% isoflurane anesthesia.

### Drug administration and X-ray irradiation of mice

Ten days later, when the tumor size reached approximately 100 mm^3^, the mice were randomly divided into four groups. In accordance with a previous study by Luo et al. [27], erastin was dissolved in 5% DMSO/corn oil and intraperitoneally injected into the NCI-H1975 cell-transplanted mice at a dose of 15 mg/kg/day for 3 days at 24-h intervals. For the combination therapy, 24 h after the last erastin injection, the anesthetized NCI-H1975 cell-transplanted mice were locally irradiated with X-rays at a dose of 3 Gy (dose rate, 4.79 Gy/min).

### Statistical analysis

**Fig 1:**
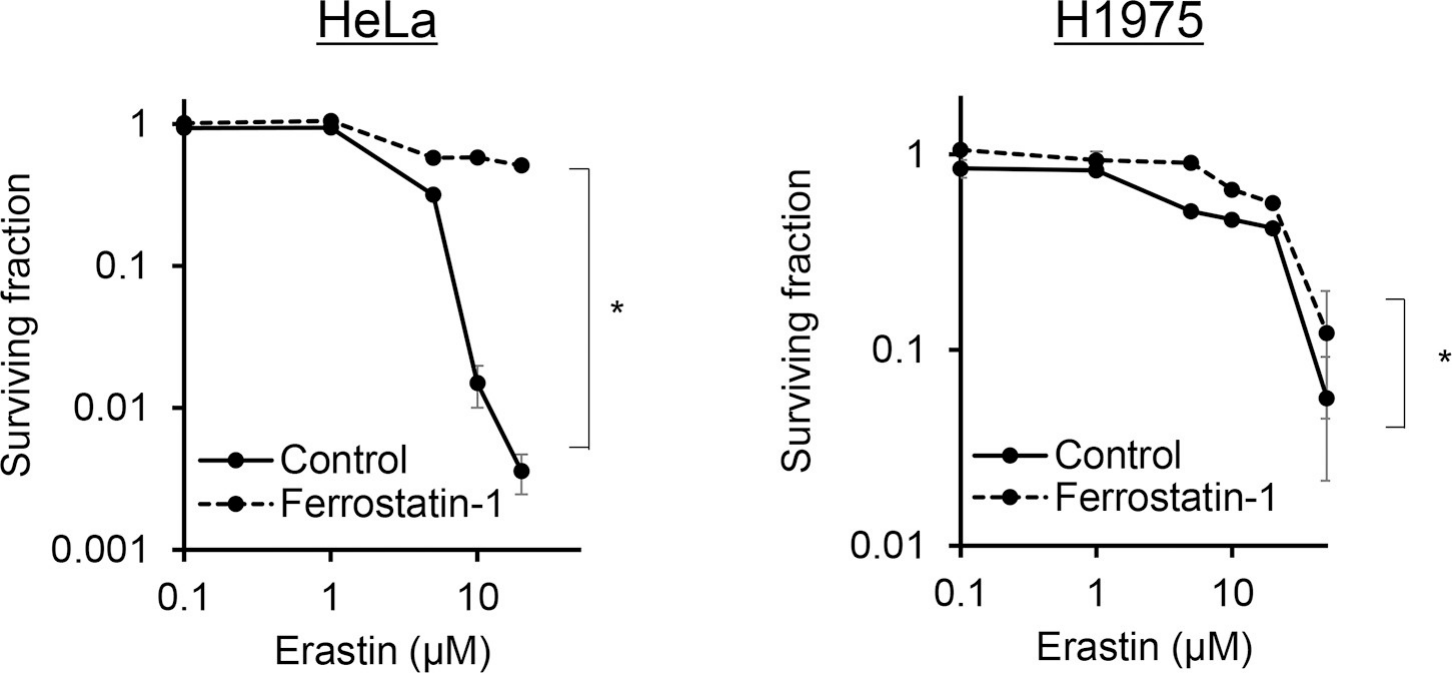
Ferrostatin-1 suppressed erastin cytotoxicity in human adenocarcinoma cell lines. Clonogenic assay of the HeLa and NCI-H1975 cells. The cells were treated with erastin alone or in combination with 1.0 μM ferrostatin-1 for 24 h. All data are expressed as mean ± S.E. (n = 3, *p < 0.0001, two-way ANOVA).

**Fig 2:**
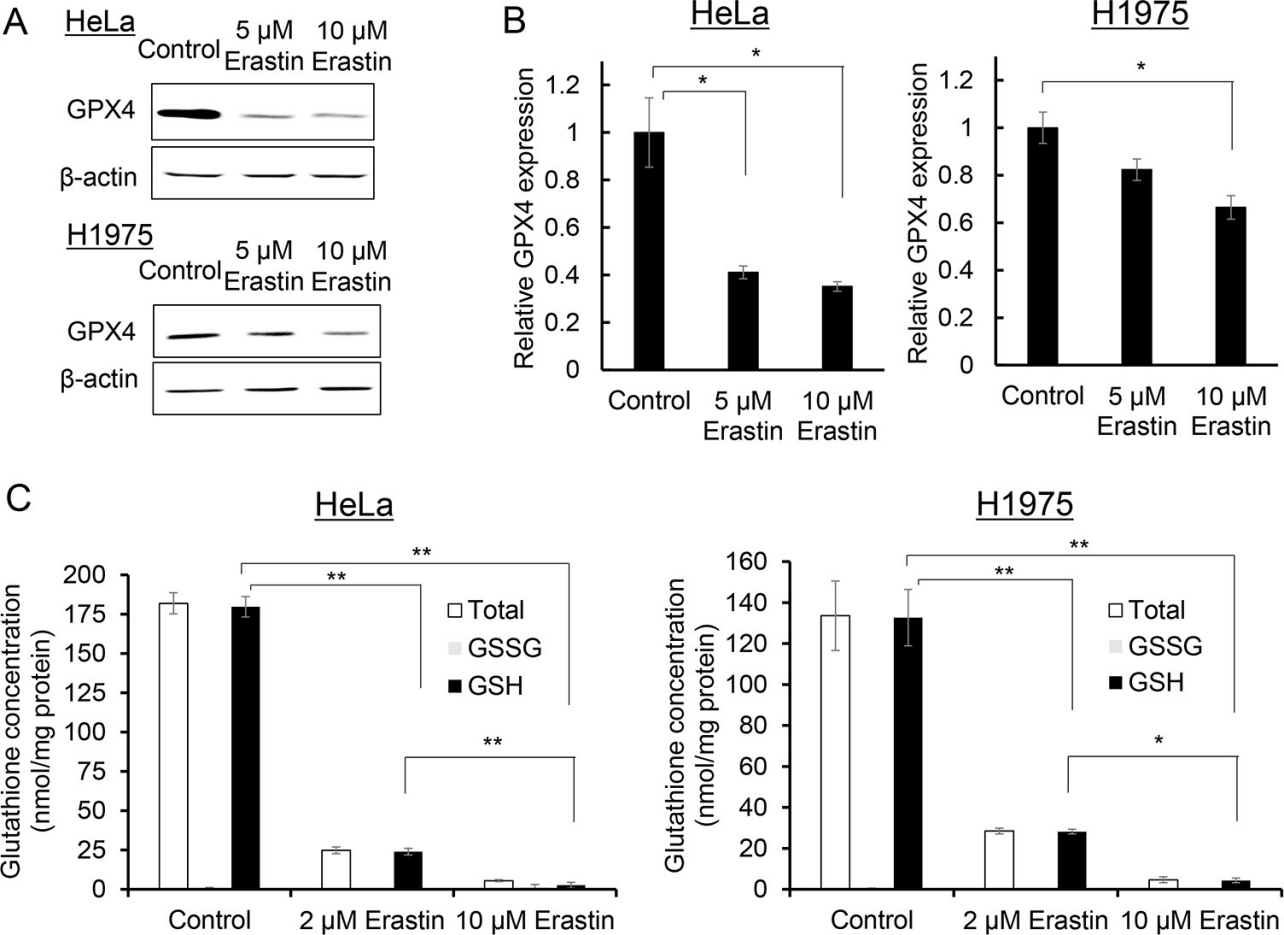
Erastin decreased GPX4 protein expression levels and intracellular glutathione concentrations in human adenocarcinoma cell lines. Western blotting of GPX4 expressions was performed on the HeLa and NCI-H1975 cells 24 h after the erastin treatment (A), and the images were analyzed to calculate the relative GPX4 expression levels in the HeLa and NCI-H11975 cells (B). The intracellular glutathione concentrations, including the total glutathione, GSH, and GSSG in the HeLa and NCI-H1975 cells, were quantified 24 h after the erastin treatment (C). All data are expressed as mean ± S.E. (n = 3, *p < 0.05, **p < 0.01, Tukey-Kramer test).

**Fig 3:**
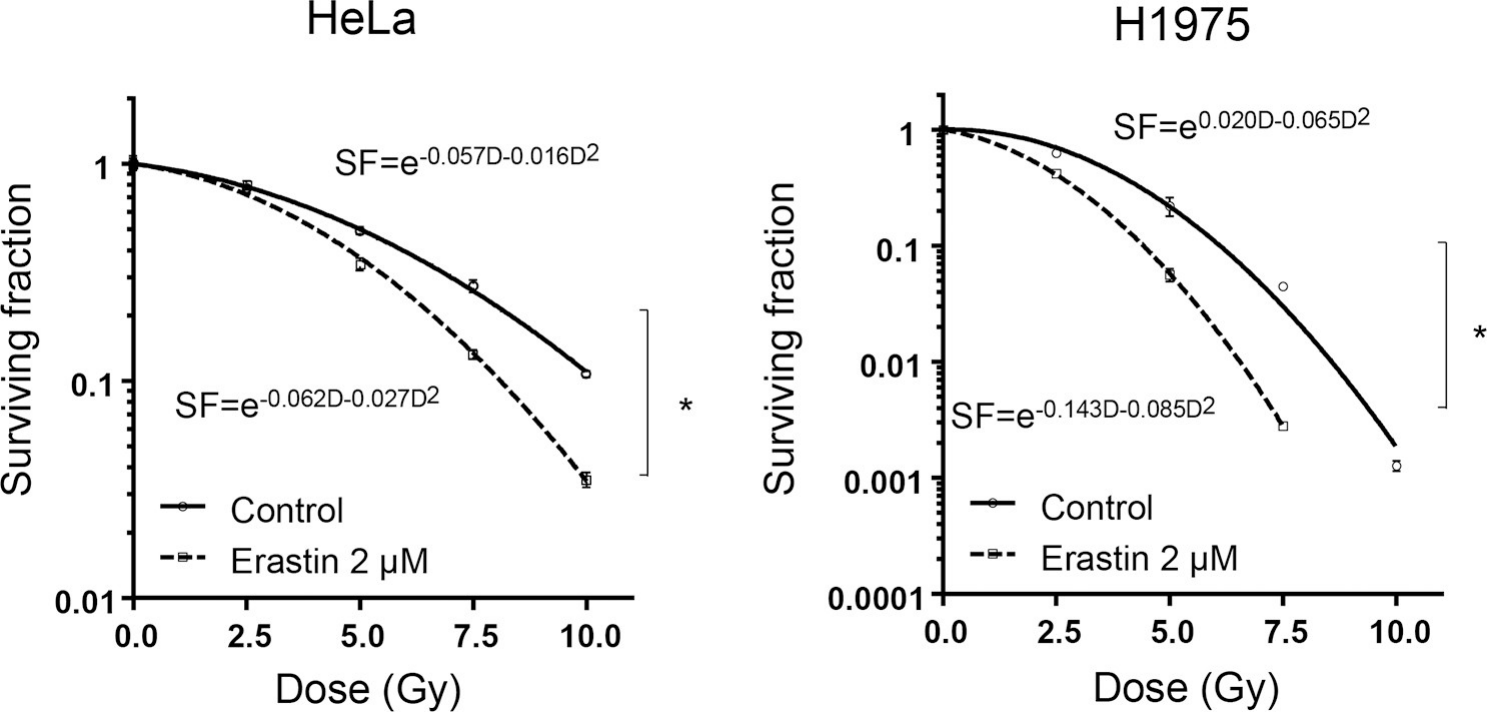
Erastin sensitized human adenocarcinoma cell lines to X-ray irradiation. Abbreviations: SF = Surviving fraction; D = Dose. The radiosensitizing effect of treatment with erastin was evaluated with a clonogenic survival assay of the HeLa and NCI-H1975 cells. Before X-ray irradiation, the cells were treated with 2 μM for 24 h. The survival curves of both the cell lines were fit into a linear quadratic (LQ) model using GraphPad Prism 7. All data are expressed as mean ± S.E. (n = 3, *p < 0.0001, two-way ANOVA).

**Fig 4:**
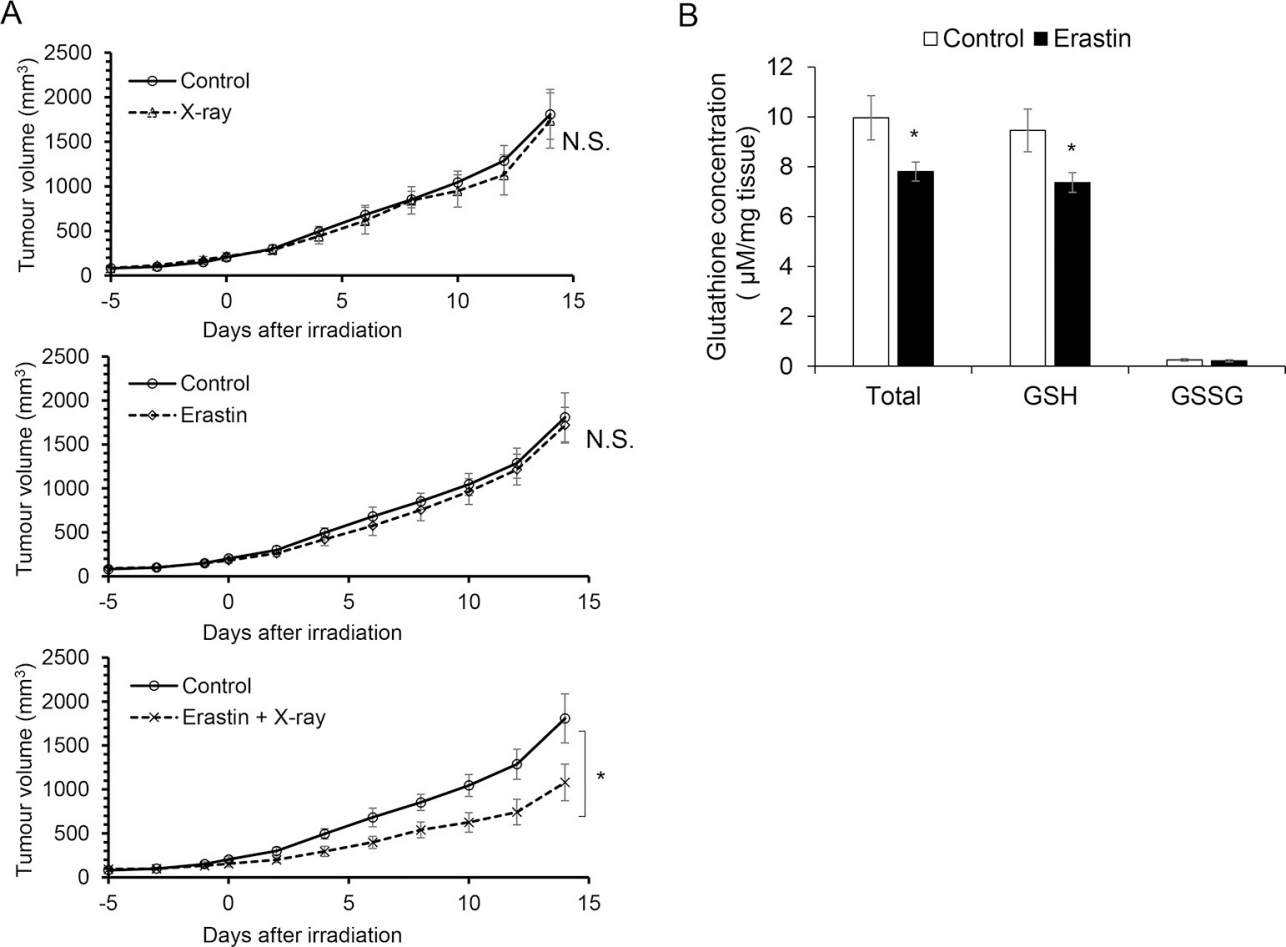
Treatment of NCI-H1975 cell-transplanted mice with erastin showed a tendency of sensitization to X-ray irradiation with a decrease in glutathione concentration. NCI-H1975 cell-transplanted mice were treated with erastin (15 mg/kg intraperitoneally) for 3 days at 24-h intervals and irradiated with X-rays at a dose of 3 Gy. The data are expressed as mean ± S.E. A statistical analysis was performed with repeated-measures two-way ANOVA [n = 5 (Control, X-ray, Erastin), n = 6 (Erastin + X-ray), *p < 0.05] (A). The glutathione concentration was quantified in erastin-treated or untreated NCI-H1975 cell-transplanted mice. Mice were treated with erastin as described above. The data are expressed as mean ± S.E (n = 7, *p < 0.05, Student’s t-test) (B).

All results are expressed as the mean ± S.E. The statistical analysis was performed using GraphPad Prism 7. The statistical significance of erastin cytotoxicity and the inhibitory effect of ferrostatin-1 on the two cell lines were examined with two-way ANOVA (Fig 1). Multiple comparisons were performed with Tukey–Kramer test (Fig 2). The statistical significance of the therapeutic effects of erastin and the X-ray irradiation on the cancer cell lines were examined with two-way ANOVA (Fig 3). The therapeutic effects of erastin, X-ray irradiation, and combination treatment against NCI-H1975 cell-transplanted mice were statistically evaluated with repeated-measures two-way ANOVA (Fig 4A). Differences in intratumoral glutathione concentration between the control and erastin-treated groups were evaluated with Student’s t-test (Fig 4B). A *p*-value < 0.05 was considered significant.

## Results

### Erastin induces ferroptosis in the human adenocarcinoma cell lines

The toxicity of erastin and its dependence on ferroptosis in human adenocarcinoma cells derived from different organs (HeLa and NCI-H1975 cells) were examined. Erastin cytotoxicity was dose-dependent in both the HeLa and NCI-H1975 cells, and their 50% growth inhibitory concentrations were approximately 3.5 and 5 μM, respectively (Fig 1). In addition, the erastin-induced cell death was significantly inhibited by 1 μM ferrostatin-1, a specific inhibitor of ferroptosis, in both the cell lines (two-way ANOVA, *p* < 0.0001 for treatment with erastin and *p* < 0.0001 for treatment with ferrostatin-1, in both the HeLa and NCI-H1975 cells) (Fig 1).

### Erastin treatment decreases intracellular concentrations of antioxidant reagents

Fig 2A and 2B show the expression level of the GPX4 protein in the HeLa and NCI-H1975 cells incubated with erastin for 24 h. The expression levels of GPX4 in both the cancer cell lines treated with erastin were significantly lower than those in untreated cells. The intracellular GSH concentrations in the untreated HeLa and NCI-H1975 cells were 181.9 ± 3.9 and 133.6 ± 8.0 nmol/mg protein, respectively (Fig 2C). Treatment with erastin significantly reduced the total glutathione and GSH concentrations in a dose-dependent manner (Tukey-Kramer test, HeLa Control vs 2 μM Erastin *p* < 0.01, HeLa Control vs 10 μM Erastin *p* < 0.01, HeLa 2 μM Erastin vs 10 μM Erastin *p* < 0.01, NCI-H1975 Control vs 2 μM Erastin *p* < 0.01, NCI-H1975 Control vs 10 μM Erastin *p* < 0.01, NCI-H1975 2 μM Erastin vs 10 μM Erastin *p* < 0.05) in both the cell lines; total glutathione concentrations of the HeLa and NCI-H1975 cells treated with 10 μM decreased to 3.0% and 3.5%, respectively, and their GSH concentrations decreased to 1.0% and 3.2%, respectively (Fig 2C).

### Erastin enhances X-ray-induced cell death

The radiosensitizing effects of erastin on the HeLa and NCI-H1975 cells were evaluated (Fig 3). The treatment with a combination of erastin and X-ray irradiation significantly decreased the survival of both the cancer cell lines (*two-way ANOVA*, *p* < 0.0001 for treatment with erastin and *p* < 0.0001 for X-ray irradiation in both the HeLa and NCI-H1975 cells) (Fig 3). The 10% lethal doses (D_10_) for the X-irradiated HeLa cells with and without treatment with erastin were 10.24 and 8.10 Gy, respectively (sensitizer enhancement ratio [SER] = 1.27). Similarly, the D_10_ values for the X-irradiated NCI-H1975 cells with and without treatment with erastin were 6.11 and 4.42 Gy, respectively (SER = 1.38).

### Treatment with erastin potentiates radiotherapy and decreases glutathione concentration in tumor xenograft models

The group administered with both erastin treatment and radiotherapy showed significant tumor growth suppression, while the group administered with erastin or radiotherapy alone showed no tumor growth suppression (Fig 4A). The values of tumor volume (mean ± S.E) at 14 days after irradiation were 1753.84 ± 288.67 mm^3^ for Control, 1738.52 ± 309.95 mm^3^ for X-ray alone, 1719.07 ± 203.13 mm^3^ for erastin alone, and 1079.89 ± 227.84 mm^3^ for erastin + X-ray. Furthermore, a glutathione quantification assay revealed that the intratumoral glutathione concentrations in erastin-treated tumors were significantly lower than those in nontreated tumors (Fig 4B).

## Discussion

In this study, we provided new findings on the ferroptosis inducer erastin in association with cancer sensitivity to X-ray irradiation. Erastin showed dose-dependent toxicity, and ferroptosis inhibitor ferrostatin-1 partly suppressed this effect in both the human adenocarcinoma cell lines, although the inhibitory ratio in the NCI-H1975 cells was smaller than that in the HeLa cells (Fig 1). In both the cell lines, the expression level of GPX4, which is a member of the glutathione peroxidase family and plays a key role in protecting cells from oxidative damage by preventing membrane lipid peroxidation, decreased after treatment with erastin, but the NCI-H1975 cells showed a smaller decrease in the GPX4 expression level compared to the HeLa cells (Fig 2A and 2B). However, erastin even at low concentrations markedly decreased the glutathione concentration in both the cell lines (Fig 2C). The clonogenic survival assay revealed that the radiosensitivities of the HeLa and NCI-H1975 cells increased when X-ray irradiation was performed after the treatment with 2 μM erastin (Fig 3). Furthermore, in vivo investigations using NCI-H1975 cell-transplanted mouse models indicated the significant radiosensitizing effect of erastin accompanied by a decrease in the tumor glutathione concentration (Fig 4).

Erastin was discovered as a direct xCT inhibitor [17, 28, 29]. Since glutathione synthesis is regulated by the cellular uptake of cysteine, inhibition of xCT leads to the suppression of GSH synthesis [29, 30]. Erastin is also implicated in iron absorption and accumulation, which results in the synthesis of ROS and lipid peroxidase [18, 31]. Thus, the iron-dependent cell death is considered to be induced by reduction in antioxidants and accumulation of free radicals. The association between antioxidants, such as glutathione or glutathione peroxidase, and tumor therapy resistance has been studied for a decade [32–35], showing that glutathione depletion decreases the radio- and chemotherapy resistance of breast cancers and glioma cells, which indicates the potential of erastin as a radiosensitizer. GPX4 is a key regulator for ferroptosis. Its major function is to reduce lipid-peroxides using GSH as a substrate. In addition, GPX4 plays a role in DNA damage repairing by reducing thymidine peroxides [36].

The present study revealed that the radiosensitizing effect of erastin in the two human adenocarcinoma cell lines and its dependence on the depletion of GSH levels by erastin, although, there were several differences between these cell lines. In the erastin cytotoxicity assay, the inhibitory effect of ferrostatin-1 was lesser on the NCI-H1975 cells than on HeLa cells. This finding indicates that treatment with erastin did not induce ferroptosis strongly in NCI-H1975 cells compared to HeLa cells. The difference in the suppression levels of GPX4 protein expression between NCI-H1975 and HeLa cells after treatment with erastin also supports this finding. Nevertheless, in vitro and in vivo experiments showed the radiosensitizing effect of erastin on both the adenocarcinoma cell lines. Contrary to the GPX4 protein expression, the intracellular and tumor glutathione concentrations in both the adenocarcinoma cell lines markedly decreased after the treatment with erastin. Thus, decrease in glutathione concentrations is the key factor sensitizing cancer cells to X-ray irradiation. Further, SER of erastin was higher in the NCI-H1975 cells despite the high basal GSH concentration in the HeLa cells. These differences were probably caused by the influence on iron metabolism. However, the intracellular iron measurement by ICP-AES and western blot analysis for TfR1 protein of the HeLa and NCI-H1975 cells revealed that treatment with erastin did not show any significant influence on the iron metabolism of either cell line (S1 Fig). Reduction in the intracellular GSH concentration causes radiosensitizing effect on several cancer cell lines [34, 35, 37, 38]. However, some studies showed no correlation between basal GSH concentration and radiosensitivity of cancer cell lines [39–41]. Thus, the difference in radiosensitivities of the HeLa and NCI-H1975 cells revealed in the present study may be caused by not only the GSH concentration but also other genetic backgrounds. The mutation status of epidermal growth factor receptor is considered to affect the radiosensitivity of non-small cell lung carcinoma, including the NCI-H1975 cells [42–44].

Compared to an in vitro GSSG/GSH quantification study (Fig 2C), an in vivo study has shown a relatively weak effect on GSH reduction (Fig 4B). The reason for this can be explained by the pharmacokinetics of erastin because erastin has low solubility, and recent studies showed a poor metabolic stability in a mouse liver microsome assay [45]. Consequently, we conducted a preliminary in vivo study to determine the suitable timing for treatment with erastin. Based on it, we selected a 3-day erastin administration protocol, which was the most effective treatment course (data not shown).

Compounds other than erastin can induce ferroptosis, which we used in this study. xCT inhibitors, such as sulfasalazine, glutamine, and sorafenib, can induce both ferroptosis and glutathione depletion [46]. Thus, in addition to erastin, these ferroptosis inducers may have a radiosensitizing effect on cancer cells. However, all ferroptosis inducers cannot sensitize cancer cells to X-ray irradiation. Different types of ferroptosis inducers such as RSL3 and DPI compounds, were identified as inhibitors of GPX4 [45]. Since these compounds can induce ferroptosis without glutathione depletion, they may not have a radiosensitizing effect similar to erastin. However, there have been only a few studies on the correlation between X-ray irradiation and these compounds, and further investigations are required to verify this hypothesis.

In our previous investigation using another xCT inhibitor, sulfasalazine, we demonstrated that the pretreatment with sulfasalazine decreased intratumoral glutathione concentration, induced a high frequency of cellular DNA damage indicated by γ-H2AX staining, and an enhanced susceptibility to radiotherapy in mouse melanoma [47]. Sulfasalazine is also a ferroptosis inducer [17], and the results obtained in this study using erastin correspond to those of our previous study. A more recent study has shown that xCT inhibition induced by erastin sensitized the breast cancer cell lines to gamma radiation [48]. In addition, the results of the present study showed a radiosensitizing effect on the cervical and lung adenocarcinoma cell lines, indicating that the combination of treatment with erastin and radiotherapy is applicable to a wide range of cancer types. Moreover, our investigation was designed for X-ray irradiation with a linear accelerator. In clinical use, linear accelerators have the advantage over cobalt-60 machines owing to their wide range of applications in cancer therapy and ease of use [49]. Therefore, our data on the radiosensitizing ratio can be easily extended to clinical studies. Moreover, recent studies showing that erastin sensitizes cancer cells to gamma radiation have strengthened our findings.

In conclusion, the present study indicates the novel potential of ferroptosis-inducing agents as radiosensitizing drugs. Furthermore, considering previous studies showing successful treatment with ferroptosis-induced cancer therapies in combination with chemotherapies, further advancements of ferroptosis-induced cancer therapies are expected.

## Supporting information

S1 Text Supplementary materials and methods. (DOCX)

S1 Fig Erastin treatment did not affect intracellular iron concentration and TfR1 protein expression level in neither HeLa and NCI-H1975 cells. Intracellular iron concentration of HeLa and NCI-H1975 cells were measured by ICP-AES. Both cells were treated with erastin for 24 h (A). Western blot analysis of TfR1 protein expressions was performed on HeLa and NCI-H1975 cells after 24 h erastin treatment (B), and the images were analyzed to calculate the relative TfR1 protein expression levels in both cells (C). All the data are presented as mean ± S.E. (n = 3)
(TIF)

S1 File Raw images. (PDF)

S2 File ARRIVE checklist. (PDF)
