# Supplementary material for: Erastin, a ferroptosis-inducing agent, sensitized cancer cells to X-ray irradiation via glutathione starvation in vitro and in vivo
Source: PLoS One. 2019 Dec 4;14(12):e0225931. doi: 10.1371/journal.pone.0225931 (PMC6892486; doi:10.1371/journal.pone.0225931)
Supplement: S1 Text — (DOCX) [file pone.0225931.s001.docx]

**Supplementary materials and methods**

**Reagents**

The following antibody was used for western blotting to detect TfR1 protein expression in HeLa and NCI-H1975 cells: anti-TfR1 (Cat. No. ab84026, Abcam).

**Measurement of intracellular iron**

The concentration of intracellular iron was determined by multiple inductively coupled plasma atomic emission spectrometry (ICP-AES) (ICPE-9000, Shimadzu, Tokyo, Japan) according to Albanese and others [1]. HeLa and NCI-H1975 cells were seeded in 150 mm dishes 16h before erastin treatment. After 24 h erastin (0 – 10 μM) treatment, cells were trypsinized and the number of cells were counted. Cells were centrifuged at 200×*g*, 3 min at 4°C and washed with PBS for twice. Samples were keep stored at -80°C deep freezer. On the day of the ICP-AES analysis, cell samples were lysed with 600 μL 60% ultra-high purity nitric acid (KANTO CHEMICAL Co., Inc., Tokyo, Japan) and incubated for 30 min at 70°C. Samples were then chilled with ice for 1 min and diluted with 8,400 μL Milli-Q water (MerkMillipore) before sample injection to ICPE-9000. In order to calculate iron concentration, iron standard solution (AccuStandard Inc., New Haven, CT) was prepared at 5, 10, 50, 100, 500 ppm.

**References**

1. Albanese A, Tsoi KM, Chan WCW. Simultaneous Quantification of Cells and Nanomaterials by Inductive-Coupled Plasma Techniques. Journal of Laboratory Automation. 2013;18(1):99-104.
