# Supplementary figures and images for: Erastin, a ferroptosis-inducing agent, sensitized cancer cells to X-ray irradiation via glutathione starvation in vitro and in vivo
Source: PLoS One. 2019 Dec 4;14(12):e0225931. doi: 10.1371/journal.pone.0225931 (PMC6892486; doi:10.1371/journal.pone.0225931)

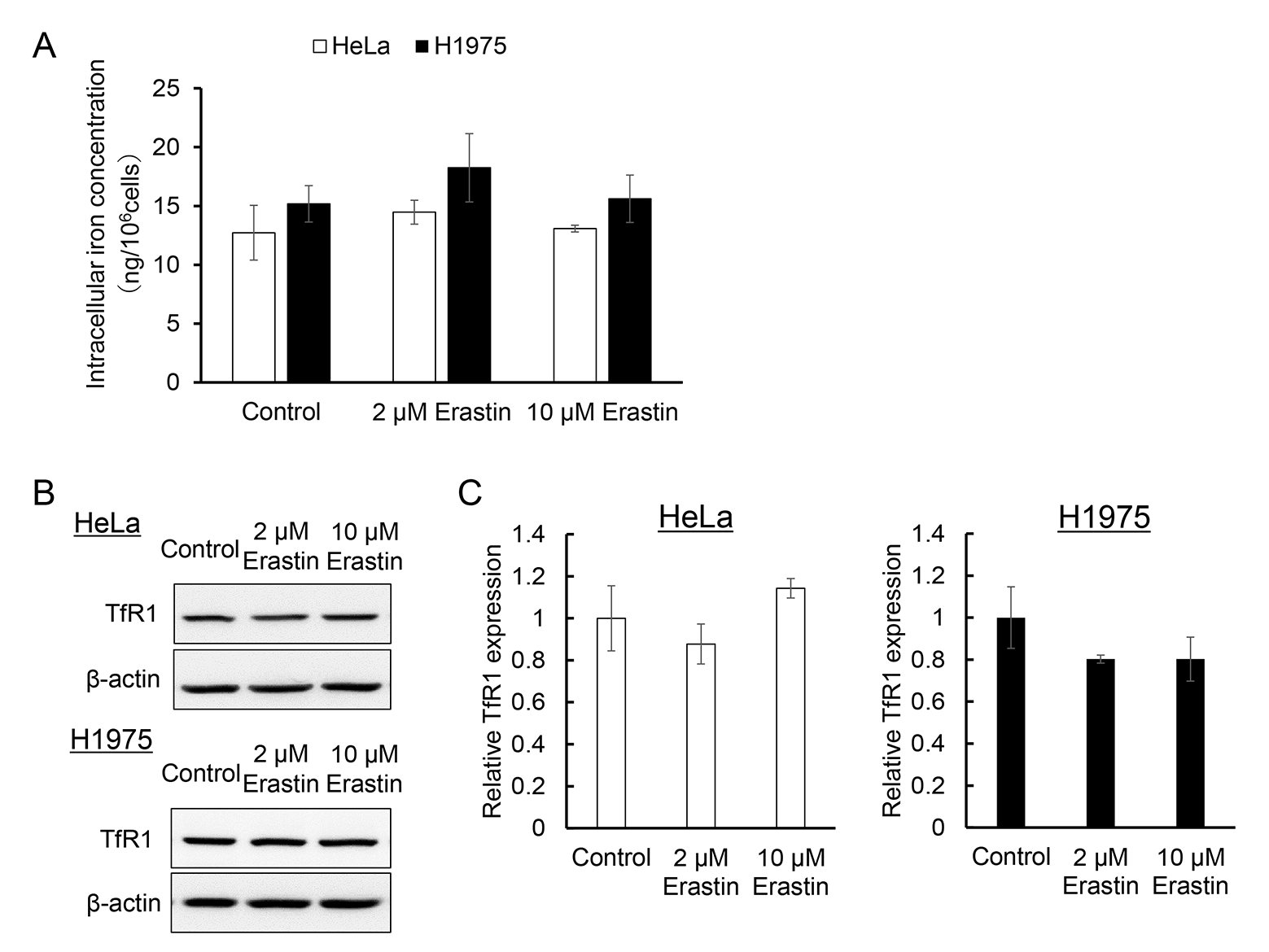

Supplement: S1 Fig — Intracellular iron concentration of HeLa and NCI-H1975 cells were measured by ICP-AES. Both cells were treated with erastin for 24 h (A). Western blot analysis of TfR1 protein expressions was performed on HeLa and NCI-H1975 cells after 24 h erastin treatment (B), and the images were analyzed to calculate the relative TfR1 protein expression levels in both cells (C). All the data are presented as mean ± S.E. (n = 3) (TIF) [file pone.0225931.s002.tif]
