# Supplementary material for: Erastin, a ferroptosis-inducing agent, sensitized cancer cells to X-ray irradiation via glutathione starvation in vitro and in vivo
Source: PLoS One. 2019 Dec 4;14(12):e0225931. doi: 10.1371/journal.pone.0225931 (PMC6892486; doi:10.1371/journal.pone.0225931)
Supplement: S1 File — (PDF) [file pone.0225931.s003.pdf]

HeLa

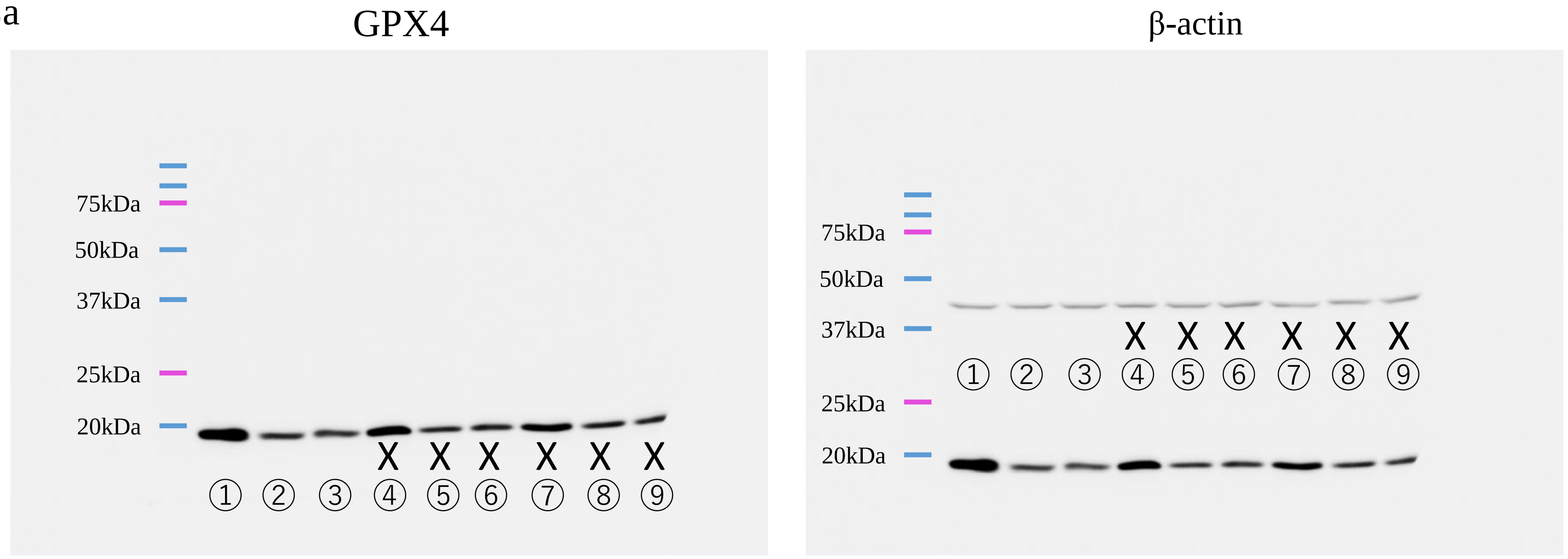

①,④,⑦ : Control ②,⑤,⑧ : 5 μM Erastin ③,⑥,⑨ : 10 μM Erastin

NCI-H1975

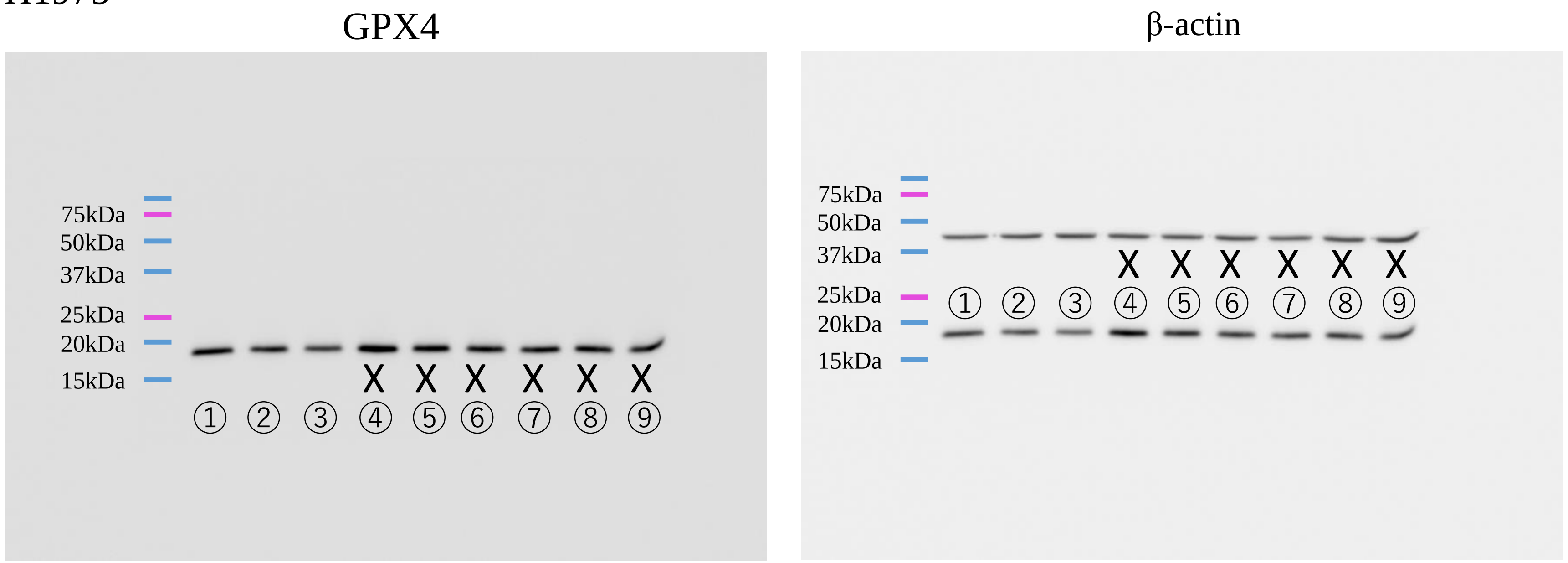

①,④,⑦ : Control ②,⑤,⑧ : 5 μM Erastin ③,⑥,⑨ : 10 μM Erastin

HeLa

TfR1

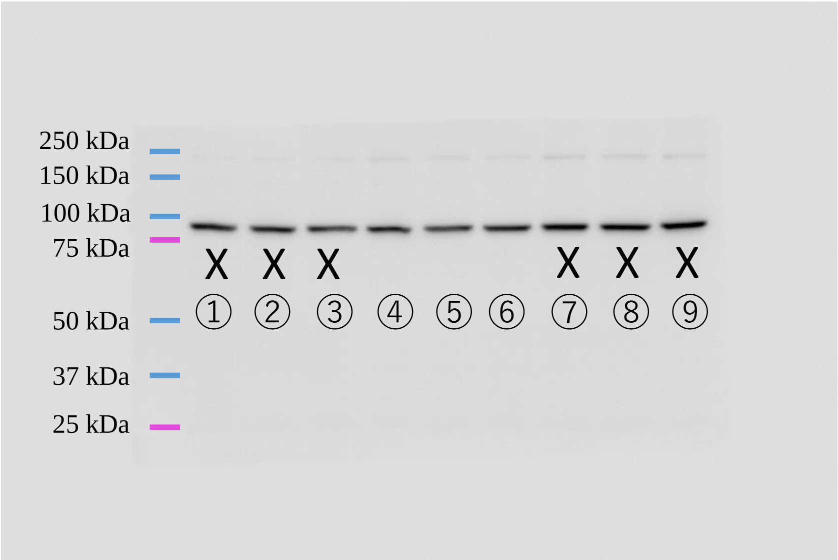

β-actin

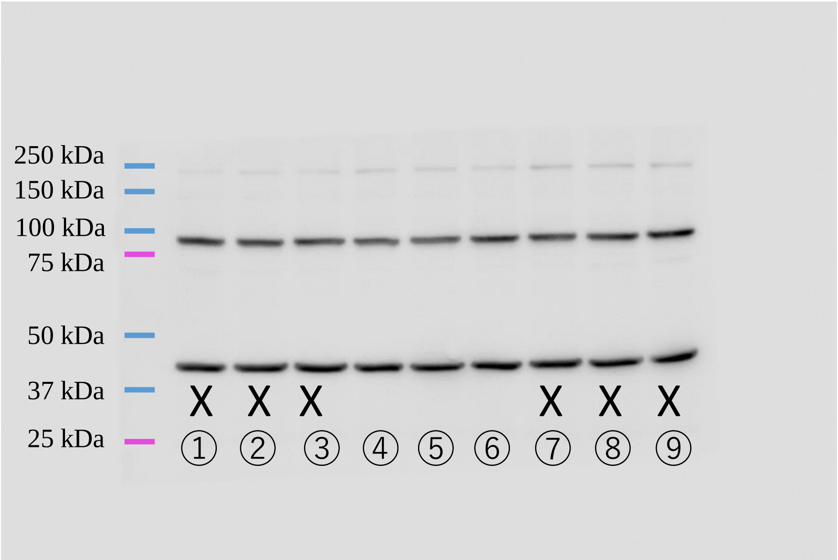

①,④,⑦ : Control ②,⑤,⑧ : 2 μM Erastin ③,⑥,⑨ : 10 μM Erastin

NCI-H1975

TfR1

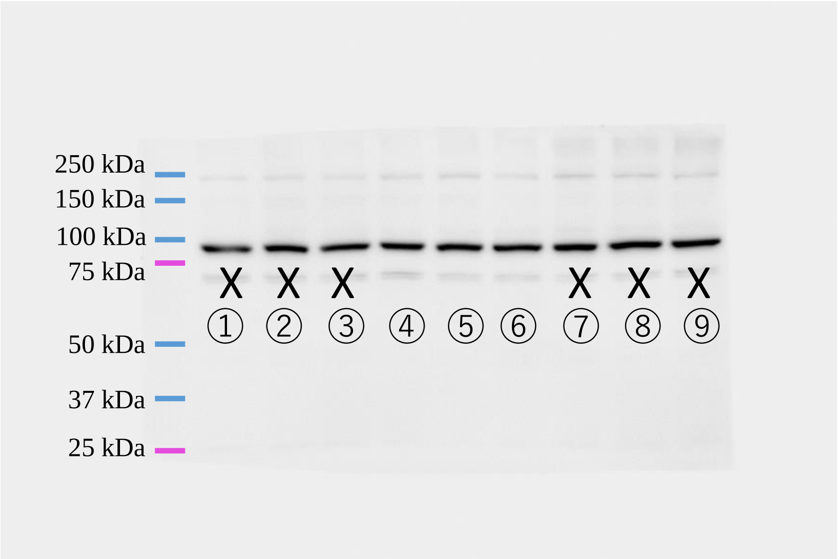

β-actin

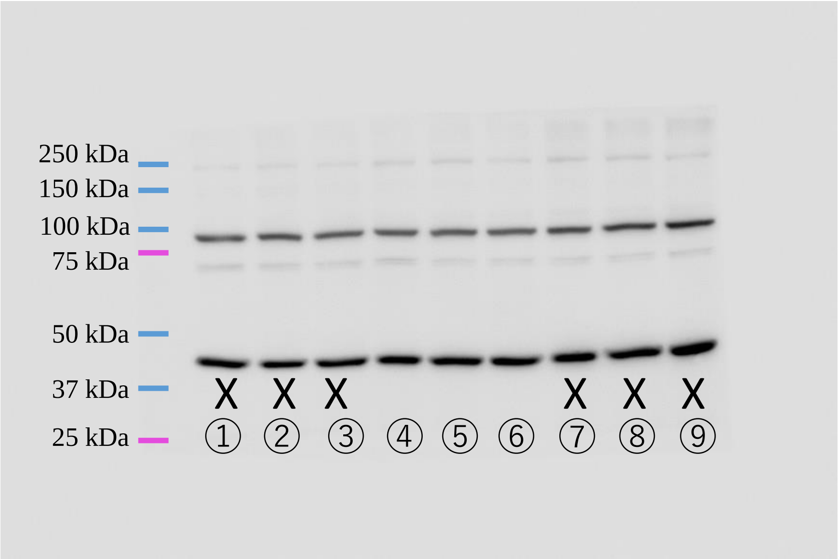

①,④,⑦ : Control ②,⑤,⑧ : 2 μM Erastin ③,⑥,⑨ : 10 μM Erastin
